# Vaginal bleeding in early pregnancy at a regional-level emergency department in KwaZulu-Natal

**DOI:** 10.4102/jcmsa.v3i1.162

**Published:** 2025-02-25

**Authors:** Mthunzi Maseko, Seelan Pillay, Jaykumaran Govender

**Affiliations:** 1 Department of Emergency Medicine, Faculty of Health Sciences, University of KwaZulu-Natal, Durban, South Africa

**Keywords:** vaginal bleeding, early pregnancy, miscarriage, ectopic, rural, emergency department

## Abstract

**Background:**

Vaginal bleeding in early pregnancy (VBIEP) affects between 15% and 25% of all pregnancies worldwide and it portends life-threatening gynaecological emergencies. One of the core functions of an emergency medicine physician is to recognise critical illness and institute high-quality emergency care. This study aimed to describe the demographics, clinical presentation, management and outcomes of patients with VBIEP presenting to the emergency department (ED).

**Methods:**

Retrospective descriptive data were collected on all patients presenting to the ED of General Justice Gizenga Mpanza Regional Hospital (GJGMRH) with VBIEP between 01 January 2022 and 30 April 2022.

**Results:**

The most affected group comprised multiparous women, in the first trimester of pregnancy between the ages of 25 and 29 year. Among HIV-positive participants majority (40.9%) were aged 35–39 years of age. Majority (87%) of patients had received no prior antenatal care. Incomplete miscarriage was the most (46.1%) common diagnosis made in the ED. Most (61.7%) patients from the ED required admission to the hospital for further care.

**Conclusion:**

This study showed that multiparous women in their first trimester of pregnancy are at highest risk of VBIEP. It also highlighted early antenatal care and early use of point-of-care ultrasound may prevent and improve outcomes with VBIEP. Incomplete miscarriage was the commonest cause. A standardised management protocol for these patients should be developed, however larger studies must be conducted to validate this.

**Contribution:**

This study contributes to the limited literature on VBIEP in low to middle-income countries and the findings may influence the department of health’s allocation of resources to this condition.

## Introduction

Vaginal bleeding in early pregnancy (VBIEP) affects 15% – 25% of all pregnancies worldwide.^1,2^ Vaginal bleeding in early pregnancy contributes significantly to the burden of maternal deaths and morbidity.^3^ Pregnancy-related haemorrhage is the third leading cause of maternal mortality in South Africa (SA).^4^ Vaginal bleeding in early pregnancy is associated with early pregnancy loss, an increased risk of ante-partum haemorrhage in late pregnancy, premature rupture of membranes and an increased risk of adverse foetal outcomes.^5^ In dealing with pregnancy-related complications such as VBIEP, one of the key areas highlighted in the Saving Mothers Report of 2019 was the challenge of poorly skilled healthcare providers in district hospitals and overburdened services at regional and tertiary hospitals.^4^

Emergency departments and gynaecology departments (GDs) internationally, have published substantial literature about VBIEP.^3,6,7^ However, there is a paucity of data among women with VBIEP in low- to middle-income countries (LMICs). It has been consistently documented in multiple studies that the age group at highest risk for VBIEP is between 20 and 29 years.^1,6^ Few studies have shown that multiparous women are particularly more vulnerable to VBIEP.^8,9^

The aetiologies of VBIEP are well-known. They include the spectrum of miscarriage, which can be further divided into threatened, inevitable, incomplete and complete miscarriage. Other causes of VBIEP are ectopic pregnancy, uterine pathology such as gestational trophoblastic diseases, subchorionic haemorrhage as well as cervical and vaginal lesions.^10^ The VBIEP can range from spotting to heavy vaginal bleeding which is defined as having to change pads at least twice an hour for two or more hours.^11^ There is a growing trend in the literature of utilising the shock index (SI) to evaluate the bleeding obstetric patient and various studies have defined a positive SI as above 0.85 in early pregnancy.^12^ These patients are at risk of developing anaemia.^13^ According to the World Health Organization (WHO) anaemia can be classified as mild (10 g/dL – 10.9 g/dL), moderate (7 g/dL – 9.9 g/dL) and severe (< 7 g/dL).^14^

Emergency Medicine (EM) is a relatively new discipline in SA.^15^ An EM specialist’s core function is to provide early assessment and recognition of critical illness and institute high-quality emergency care. Vaginal bleeding in early pregnancy is an emergency that commonly presents to the EDs with developed countries managing between 110 000 and 500 000 cases annually.^16,17^ There is currently insufficient evidence describing the estimated number of these cases in SA. Vaginal bleeding in early pregnancy is a cause for concern among many patients and its occurrence requires adequate and timely action of a duly trained multidisciplinary team to manage it.^18^ This study will aim to describe the demographics, clinical presentation, management and outcomes in VBIEP.

## Research methods and design

The research was conducted at the General Justice Gizenga Mpanza Regional Hospital (GJGMRH), which serves as the sole regional hospital in the public sector in KwaDukuza municipality in the Ilembe district with an estimated 350 000 females.^19^ The emergency department (ED) at GJGMRH accepts referrals from nine district clinics, two community health centres, three district hospitals, general practitioners and self-referrals.

This study was a retrospective chart review of patients seen in the ED of GJGMRH between 01 January 2022 and 30 April 2022.

A convenience sampling strategy was used and cases for inclusion were identified from the ED triage registry and the medical records were requested from the medical registry. The gestational age was determined using the last normal menstrual period or estimation by early ultrasound.

### Inclusion criteria

Pregnant females presenting to the ED with VBIEP at less than 25 weeks gestational age, as per local institutional policy were included in this study.

### Exclusion criteria

Females with pregnancy whose gestation was more than 25 weeks and patients with induced termination of pregnancy were excluded from this study. Patients’ files that are duplicates or have incomplete data were also excluded.

### Data analysis

Descriptive statistics, such as frequency, proportions, mean and standard deviations were used to summarise the data. A biostatistician analysed the data using version 18 of Stata package for statistical analysis.

### Ethical considerations

Ethical approval was granted by the University of KwaZulu-Natal Biostatistics Research Ethics Committee (BREC reference: BREC/00005175/2023) and the KwaZulu-Natal Department of Health (National Health Research Database [NHRD] reference: KZ_202306_013).

## Results

During the study period, 3849 patients were seen in the ED. Of these, 151 (4%) patients were identified as having VBIEP. The number of patients who made it into the final analysis was 115. A total of 36 patient records were excluded, one patient had a pregnancy over the gestation of 25 weeks, 12 files had insufficient data, six patients had VBIEP as a result of termination of pregnancy and 17 files could not be found at the medical registry.

A total of 115 women between the ages of 16 and 42 years were included, with a median age of 27 years (interquartile range [IQR]: 23–33), Table 1. Nulliparous and multiparous patients accounted for 43 out of 115 (37.4%) and 72 out of 115 (62.6%) of the cases, respectively. Unbooked patients who had received no prior antenatal care accounted for 100 out of 115 (87%). The median gestational age was 10 weeks (IQR: 5–14). Heavy vaginal bleeding and the presence of lower abdominal pain (LAP) was observed in 63 out of 115 (54.8%) and 84 (73%) of the study group, respectively.

**TABLE 1:** Socio-demographic and obstetric characteristics of women presenting with vaginal bleeding in early pregnancy to a regional-level Emergency Department in KwaZulu-Natal, 01 January 2022 – 30 April 2022 (*N* = 115).

Variable(s)	Total	
	*n*	%
**Age group (years)**		
15–19	14	12.2
20–24	21	18.3
25–29	34	29.6
30–34	24	20.9
35–39	21	18.3
40+	1	0.9
**Parity**		
0	29	25.2
1	43	37.4
2	29	25.2
3+	14	12.2
**Booked for ANC**		
No	100	87.0
Yes	12	10.4
Unspecified	3	2.6
**Gestational age**		
Gestational age ≤ 12 weeks	82	71.3
Gestational age > 12 weeks	33	28.2
**Determination of gestational Age**		
Use of the LNMP	85	73.9
Use of early ultrasound	30	26.1
**Previous caesarean section**		
0	104	90.4
1	8	7.0
2	3	2.6
**Previous miscarriage**		
0	94	81.7
1	17	14.7
≥ 2	4	3.6
**Previous ectopic**		
0	0	0.0
**Type of vaginal bleed**		
Heavy bleed	63	54.8
Spotting	31	27.0
Unspecified	21	18.3
**Lower abdominal pain**		
Present	84	73.0
Absent	31	27.0
**Referral**		
Clinic	66	57.4
District hospital	6	5.2
General practitioner	12	10.4
Self-referral	31	27.0

ANC, ante-natal care; LNMP, last normal menstrual period.

**FIGURE 1:**
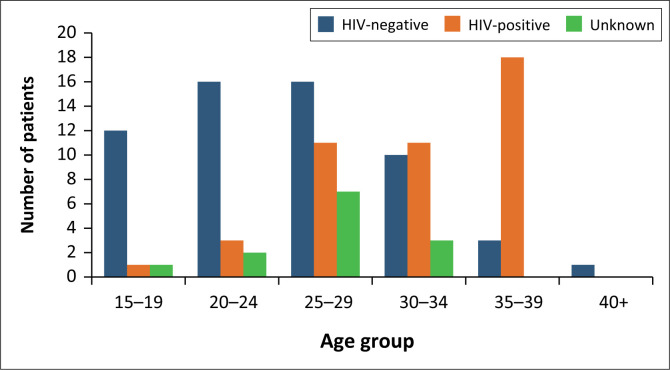
Comparison of human immunodeficiency virus status by age group of women with early pregnancy vaginal bleeding presenting to an emergency department in the KwaZulu-Natal, South Africa, 01 January 2022 – 30 April 2022.

Out of the 115 participants, 58 out of 115 (50%) patients were HIV-negative, while HIV-positive patients accounted for 44 out of 115 (38.3%) and 13 out of 115 (11.3%) were HIV-unknown. Among the HIV-positive patients, 18 out of 44 (40.9%) were between the ages of 35 and 39 years, followed by equal proportions 11 out of 44 (25.0%) in the 30–34 years and 25–29 years age groups (Figure 1). Human immunodeficiency virus-positive patients accounted for seven out of 13 (53.8%) of all ectopic pregnancies, which had hospital stay of greater than 72 h.

The median systolic blood pressure (SBP) was 113 mmHg (IQR: 105–121) and the average heart rate (HR) was 95 beats per minute (bpm). The mean SI was calculated at 0.86. The median haemoglobin (Hb) level was 10.8 g/dL (IQR: 4.3–12.2), and 60 out of 115 (52.1%) were anaemic on presentation. The rhesus (Rh) status was known in 10 out of 115 (8.7%) participants (Table 2). Urine dipstick testing was performed on all patients and it was found that 62 out of 115 (53.9%) had haematuria.

**TABLE 2:** Clinical findings of women presenting with vaginal bleeding in early pregnancy to a regional-level Emergency Department in KwaZulu-Natal, 01 January 2022 – 30 April 2022 (*N* = 115).

Variable(s)	Total	
	*n*	%
**Haemodynamic variables**		
Systolic Blood Pressure ≥ 90 mmHg	104	90.4
Systolic Blood Pressure < 90 mmHg	11	9.6
Heart rate > 100 bpm	75	65.2
Heart rate ≤ 100 bpm	40	34.8
**Shock Index (SI)**		
Positive SI	21	18.3
Negative SI	94	81.7
**Haemoglobin (Hb)**		
Normal	55	47.8
Mild anaemia	24	20.8
Moderate anaemia	26	22.6
Severe anaemia	10	8.7
**Rhesus status**		
Positive	9	7.9
Negative	1	0.9
Unknown	105	91.2
**Urine dipstick**		
Blood	62	53.9
Leukocytes and/or nitrites	14	12.1
No abnormality	39	34.0

In the study sample blood and blood products transfusions in the ED were administered to 16 out of 115 (13.9%), Table 3. Antibiotics were prescribed in 11.3% (*n* = 13/115) of cases. Analgesia was given to 65 out of 115 (56.5%) patients. None, 0 out of 115 (0%), of the patients were administered anti-D immunoglobulin in the ED. Oxytocin was prescribed to 12.2% (*n* = 14/115) of patients. Emergency department point-of-care ultrasound (POCUS) was performed in 9.6% (*n* = 11/115) of patients.

**TABLE 3:** Interventions performed in the emergency department on woman with vaginal bleeding in early pregnancy presenting to a regional-level Emergency Department in KwaZulu-Natal, SA, 01 January 2022 – 30 April 2022 (*N* = 115).

Variable(s)	Total	
	*n*	%
**Blood transfusion**		
No blood given	99	86.0
Normal haemoglobin	1	0.9
Mild anaemia	1	0.9
Moderate anaemia	6	5.2
Severe anaemia	8	7.0
**Intravenous fluid administration**		
No	44	38.3
Yes	71	61.7
**Antibiotics**		
None given	102	88.7
Ceftriaxone and metronidazole	6	5.2
Amoxicillin and clavulanic acid	4	3.4
Gentamicin and metronidazole	2	1.8
Ampicillin	1	0.9
**Analgesia type**		
None given	50	43.5
Ibuprofen and paracetamol	7	6.1
Fentanyl	5	4.3
Morphine	5	4.3
Paracetamol only	46	40.0
Diclofenac	1	0.9
Tramadol	1	0.9
**Anti-D immunoglobulin administration**		
No	115	100.0
Yes	0	0.0
**Oxytocin administration**		
No	101	87.8
Yes	14	12.2
**POCUS findings**		
Not performed	104	90.4
Free fluid in the abdomen	6	5.2
Intra uterine pregnancy	2	1.7
No foetal heart activity	3	2.6

POCUS, point-of-care ultrasound.

**FIGURE 2:**
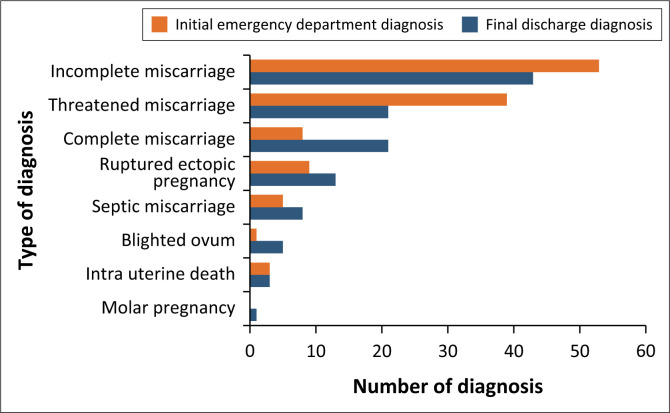
Initial and final diagnoses of women with early pregnancy vaginal bleeding presenting to an emergency department in the KwaZulu-Natal, South Africa, 01 January 2022 – 30 April 2022.

Incomplete miscarriages accounted for 53 out of 115 (46.1%) of initial ED diagnoses as a cause of VBIEP and 43 out of 115 (37.4%) as the final discharge diagnosis, Figure 2. Ruptured ectopic pregnancy accounted for 13 out of 115 (11.3%) of the final discharge diagnosis.

Patients who were admitted requiring in-patient care and monitoring amounted to 71 out of 115 (61.7%) and 41 out of 115 (35.7%) were discharged directly from the ED (Table 4). Manual vacuum aspiration (MVA) and abdominal laparotomy were definitive management strategies in 42 out of 115 (36.5%) and 12 out of 115 (10.4%) patients, respectively. Patients who were discharged from the ED with a hospital length of stay of less than 6 h accounted for 45 out of 115 (39.4%) of the study sample.

**TABLE 4:** Outcomes of patients presenting with vaginal bleeding in early pregnancy to a regional-level Emergency Department in KwaZulu-Natal, South Africa, 01 January 2022 – 30 April 2022 (*N* = 115).

Variable(s)	Total	
	*n*	%
**Disposition from ED**		
Admitted	71	61.7
Discharged	41	35.7
Refusal of hospital treatment	3	2.6
Demised	0	0.0
**Treatment**		
Laparotomy	12	10.4
Manual vacuum aspiration	42	36.5
Medical abortifacient	1	0.9
General advice and symptomatic treatment	48	41.7
Uterine evacuation	12	10.4
**Hospital length of stay**		
< 6 h	45	39.1
7–24 h	37	32.2
25–48 h	7	6.1
49–72 h	13	11.3
> 72 h	13	11.3

ED, emergency department.

## Discussion

This study aimed to describe the demographics, comorbidities, clinical presentation, management and outcomes in VBIEP. According to our study, individuals between the ages of 24 and 29 comprise the highest number of patients experiencing VBIEP. These results align with research conducted in other regions of the world, including the United Kingdom where Everett et al. found that 31% of their study participants were aged between 25 and 29 years of age.^1^ Our findings also revealed that a majority of patients who experienced VBIEP were multiparous. A retrospective analysis of vaginal bleeding and ectopic pregnancies in a Gauteng district hospital showed that 46% of participants were multiparous women, compared to 11.8% who were nulliparous.^20^ The majority of patients with VBIEP presented in the first trimester, with a median gestational age of 10 weeks. Similar findings were reported in a study conducted in another LMIC, Nigeria, which revealed that 59.2% presented at 8–12 weeks gestation.^21^ According to our study, the majority of patients experienced heavy vaginal bleeding. This supports the final diagnosis in the majority of cases, which was incomplete miscarriage. In contrast, a study conducted by Hasan et al. in the United States (US) found that only 8% of patients had heavy bleeding and that was associated with a higher risk of miscarriage.^2^

A substantial number of patients presenting with VBIEP were found to be unbooked and lacking prior antenatal care. This observation is consistent with a survey conducted by the United Nations Children’s Emergency Fund (UNICEF), which indicates that levels of antenatal care are at their lowest in sub-Saharan Africa and Southeast Asia.^22^ This result emphasises the current state of antenatal care in these regions. Furthermore, these findings are of concern especially with the findings that Chireh et al. demonstrated that early ANC visits lower the risk of miscarriage by 43%.^23^

In 2017, The National Department of Health surveyed HIV prevalence among pregnant females attending the Ante-Natal Clinic in KwaZulu-Natal. The findings revealed that 41.1% of the participants were HIV-positive.^24^ Our study indicated a similar result of 38.3%. The age group with the highest HIV prevalence in the aforementioned survey was between 24 and 29 years, whereas our study found the highest prevalence among those aged 35–39 years. It is important to notice that 13% of the patients’ HIV statuses were unknown, which could potentially account for this variation. In this study we found a significant number of participants with ectopic pregnancies were also HIV-positive. These findings are similar to what Mokoena et al. found, 56%, and the prevailing theory is the relationship between HIV and pelvic inflammatory disease, which is a risk factor for ectopic pregnancy.^25^

In clinical practice, the diligent observation of vital signs is an essential step in providing a comprehensive assessment of a patient. Our study found that despite the majority of patients presenting with heavy vaginal bleeding, very few of them were haemodynamically unstable. In this study, the median SBP and average HR were 113 mmHg and 95 bpm. These findings are consistent with those of DeVilbilis, where they found a mean SBP of 111 mmHg.^26^

Anaemia is a common disorder affecting pregnant women in developing countries.^13^ Severe anaemia was present in 8.7% of cases and we found that these patients were diagnosed with a ruptured ectopic pregnancy, an incomplete and septic miscarriage. In this study, blood products in the ED were transfused in 14% of cases, which was in contrast to Anikwe et al. where they transfused 25% of the patients who presented with a miscarriage and Nzaumvilla et al. transfused 48% of patients presenting with ectopic pregnancies.^20,21^ In our study these differences may be accounted for by our study cohort having more haemodynamically stable patients.

Gharoro et al. found that 80.9% of their study participants had associated abdominal tenderness on physical examination.^9^ We observed that in 73% of participants LAP was a co-complaint. Emergency department medical officers (MOs) had varying analgesic prescribing practices, with acetaminophen being the most common analgesic of choice. We hypothesise that this choice is because acetaminophen has a Food and Drug Administration (FDA) rating of group B, adverse maternal or foetal effects from acetaminophen use in pregnancy have not been reported.^27^

There is a growing trend in the medical literature of EM physicians using POCUS to assist in the diagnosis of the cause of vaginal bleeding in pregnant patients. International studies have shown that performing POCUS on first-trimester patients with vaginal bleeding can significantly reduce ED wait times. Chiem et al. demonstrated that pelvic ultrasound performed by appropriately trained ED doctors reduced ED length of stay by 139 min and with comparable ultrasound examination findings compared to scans performed by the radiology department.^28^ However, our present study found a lack of utilisation of POCUS in this patient cohort, with ED MOs using it in 9.6% of cases, when they suspected a ruptured ectopic pregnancy. These findings are consistent with those of Khanyi et al., who also found a low utilisation rate of 1% of all POCUS performed on pregnant patients in the same ED.^29^

The ED serves as the initial point of contact for patients presenting with VBIEP, where a working differential diagnosis is generated to inform initial management before referral to the GD for definitive management. Incomplete miscarriages were found to be the most common cause of VBIEP in the study population, accounting for 46% of cases. The majority of patients with incomplete miscarriages were referred to the GD and were treated primarily with MVA of the products of conception, with only one patient being treated with a medical abortifacient agent during the study period. A study conducted in Central Africa by Anikwe et al. found higher rates of up to 70% of cases were incomplete miscarriages and 85% were treated with MVA.^21^

Previous studies in KwaZulu-Natal in 2000 have described septic miscarriages complicated 22% of incomplete miscarriages owing mostly to unsafe abortion practices at the time.^30^ Our study showed a significantly lower rate of cases of septic miscarriage. Antibiotics were found to be an integral part of the management of septic miscarriages, with all patients diagnosed with a septic miscarriage in the ED receiving antibiotics in contrast to a study carried out at Steve Biko Academic Hospital where 21.1% of patients with septic miscarriage did not receive antibiotics during the initial evaluation and resuscitation.^31^ Our study found that the use of antibiotics varied, with ceftriaxone and metronidazole being a common combination, which differs from the National Department of Health Standard Treatment Guidelines that recommend amoxicilin-clavulanic acid, gentamicin and ciprofloxacin.^32^ All patients with septic miscarriages were subsequently taken to the operating room (OR) for uterine evacuation as per institutional policy.

Ruptured ectopic pregnancy was responsible for a significant number of cases of VBIEP in this study. A proportion of the ruptured ectopic pregnancies were missed in the ED on the initial evaluation, four out of 13 (30.7%,) and misdiagnosed as incomplete miscarriages; however, no mortalities were recorded. Higher rates of misdiagnosis (48.7%) and mortalities from ruptured ectopic pregnancy were observed in a district hospital in Gauteng, SA.^20^ This is a concerning finding as ruptured ectopic pregnancy remains a significant contributor to maternal deaths in SA.^4^ We hypothesise that the misdiagnosed ectopic pregnancies are because of the low utilisation of POCUS in the ED for this cohort of patients.

We observed that many patients were discharged directly from the ED, with general advice and symptomatic treatment, and spent less than 6 h in the ED. The hospital length of stay of our study cohort was longer compared to an average of 2 h in high income countries (HIC) where they have dedicated early pregnancy units (EPU) that exclusively see patients with early pregnancy complications including VBIEP.^33^

### Limitations

The study’s limitations included a convenience sampling strategy and not being able to retrieve about 25% of the patient records from the medical registry. In addition, from the retrieved records some data were missing, and those files were excluded. This was a LMIC single-centre study at a regional ED, results may differ at the district or tertiary level.

## Conclusion

This study showed that multiparous women in their first trimester of pregnancy are at highest risk for VBIEP. It also highlighted early antenatal care and early use of POCUS may prevent and improve outcomes with VBIEP. Incomplete miscarriage was the commonest cause. A standardised management protocol for these patients should be developed; however, larger studies must be conducted to validate this.

## References

[1] Everett C. Incidence and outcome of bleeding before the 20th week of pregnancy: Prospective study from general practice. BMJ. 1997;315(7099):32–34. 10.1136/bmj.315.7099.32 9233324 PMC2127042

[2] Hasan R, Baird DD, Herring AH, Olshan AF, Jonsson Funk ML, Hartmann KE. Association between first-trimester vaginal bleeding and miscarriage. Obstetr Gynecol. 2009;114(4):860–867. 10.1097/AOG.0b013e3181b79796 PMC2828396 19888046

[3] Maharlouei N, Zeinab Z, Ezat M, Kamran BL. Maternal mortality rate in Fars province: Trends and associated factors in a community-based survey. Arch Iran Med. 2012;15(1):14–17. 22208437

[4] NCCEMD. Saving Mothers 2019 report: Seventh triennial report on confidential enquiries into maternal deaths in South Africa. Pretoria: DOH; 2019.

[5] Obed JY, Adewole IF. Antepartum haemorrhage: The influence of first trimester uterine bleeding. West Afr J Med. 1997;16(1):24–26. 9133819

[6] Wittels KA, Pelletier AJ, Brown DFM, Camargo CA, Jr. United States emergency department visits for vaginal bleeding during early pregnancy, 1993–2003. Am J Obstetr Gynecol. 2008;198(5):523.e1–523.e6. 10.1016/j.ajog.2007.11.011 18191797

[7] Trostian B, Curtis K, McCloughen A, et al. Experiences and outcomes of women with bleeding in early pregnancy presenting to the Emergency Department: An integrative review. Australas Emerg Care. 2021;25(1):55–83. 10.1016/j.auec.2021.04.006 34083158

[8] Harville EW. Vaginal bleeding in very early pregnancy. Hum Reprod. 2003;18(9):1944–1947. 10.1093/humrep/deg379 12923154

[9] Gharoro EP, Igbafe AA. Ectopic pregnancy revisited in Benin City, Nigeria: Analysis of 152 cases. Acta Obstetr Gynecol Scand. 2002;81(12):1139–1143. 10.1034/j.1600-0412.2002.811207.x 12519110

[10] Odendaal HJ, Gebhardt GS. Bleeding in early and late pregnancy. Curr Obstetr Gynaecol. 1999;9(2):82–87. 10.1016/S0957-5847(99)90005-7

[11] Janssen CAH, Scholten PC, Heintz APM. A simple visual assessment technique to discriminate between menorrhagia and normal menstrual blood loss. Obstetr Gynecol. 1995;85(6):977–982. 10.1016/0029-7844(95)00062-V 7770270

[12] Birkhahn RH, Gaeta TJ, Bei R, Bove JJ. Shock index in the first trimester of pregnancy and its relationship to ruptured ectopic pregnancy. Acad Emerg Med. 2002;9(2):115–119. 10.1197/aemj.9.2.115 11825835

[13] Suryanarayana R, Chandrappa M, Santhuram AN, Prathima S, Sheela SR. Prospective study on prevalence of anemia of pregnant women and its outcome: A community based study. J Fam Med Prim Care. 2017;6(4):739–743. 10.4103/jfmpc.jfmpc_33_17 PMC5848390 29564255

[14] Pasricha S-R, Rogers L, Branca F, Garcia-Casal M-N. Measuring haemoglobin concentration to define anaemia: WHO guidelines. Lancet. 2024;403(10440):1963–1966. 10.1016/S0140-6736(24)00502-6 38493792

[15] Wallis LA, Garach SR, Kropman A. State of emergency medicine in South Africa. Int J Emerg Med. 2008;1(2):69–71. 10.1007/s12245-008-0033-3 19384654 PMC2657239

[16] Hodkinson PW, Wallis LA. Cross-sectional survey of patients presenting to a South African urban emergency centre. Emerg Med J. 2009;26(9):635–640. 10.1136/emj.2008.063362 19700577

[17] Hanewinckel R, Jongman HP, Wallis LA, Mulligan TM. Emergency medicine in Paarl, South Africa: A cross-sectional descriptive study. Int J Emerg Med. 2010;3(3):143–150. 10.1007/s12245-010-0185-9 21031037 PMC2926869

[18] Ixchel Suyapa Reyes E. Obstetric Hemorrhage, its role in maternal morbidity and mortality and the importance of its diagnosis, prevention and timely management. Mex J Med Res ICSA. 2020;8(15):37–44. 10.29057/mjmr.v8i15.3906

[19] Mthintso V. Profile Ilembe District Municipality (04/52) [homepage on the Internet]. Department of Cooperative Government and Traditional Affairs; 2020 [cited 2024 Dec 21]. Available from: https://www.cogta.gov.za/ddm/wp-content/uploads/2020/11/Ilembe-September2020.pdf

[20] Nzaumvila DK, Govender I, Ogunbanjo GA. An audit of the management of ectopic pregnancies in a district hospital, Gauteng, South Africa. Afr J Prim Health Care Fam Med. 2018;10(1):1757. 10.4102/phcfm.v10i1.1757 30456972 PMC6244319

[21] Anikwe C, Ikeoha C, Obuna J, Okorochukwu B, Nnadozie U. Five-year review of cases of miscarriage in a tertiary hospital in Abakaliki, South East, Nigeria. Trop J Obstetr Gynaecol. 2019;36(3):367–372. 10.4103/TJOG.TJOG_38_19

[22] Fund UNCE. Monitoring the situation of children and women [homepage on the Internet]. Geneva: World Health Organization; 2017 [updated 2024 Jan; cited 2024 Apr 30]. Available from: https://data.unicef.org/topic/maternal-health/antenatal-care/

[23] Chireh B, Essien SK, D’Arcy C. First trimester antenatal care visit reduces the risk of miscarriage among women of reproductive age in Ghana. Afr J Reprod Health. 2021;25(1):129–137. 34077119 10.29063/ajrh2021/v25i1.15

[24] Woldesenbet SA, Kufa T, Barron P, et al. Assessment of readiness to transition from antenatal HIV surveillance surveys to PMTCT programme data-based HIV surveillance in South Africa: The 2017 Antenatal Sentinel HIV Survey. Int J Infect Dis. 2020;91:50–56. 10.1016/j.ijid.2019.11.005 31712090 PMC8767461

[25] Mohosho MM. The association between the prevalence of HIV infection and ectopic pregnancy. Afr J Reprod Health. 2023;27(2):87–91. 10.29063/ajrh2023/v27i2.9 37584943

[26] Devilbiss EA, Naimi AI, Mumford SL, et al. Vaginal bleeding and nausea in early pregnancy as predictors of clinical pregnancy loss. Am J Obstetr Gynecol. 2020;223(4):570.e1–570.e14. 10.1016/j.ajog.2020.04.002 PMC7994023 32283071

[27] Collins E. Maternal and fetal effects of acetaminophen and salicylates in pregnancy. Obstet Gynecol. 1981;58(5 Suppl):57s–62s. 7031542

[28] Chiem AT, Chan CH-Y, Ibrahim DY, et al. Pelvic ultrasonography and length of stay in the ED: An observational study. Am J Emerg Med. 2014;32(12):1464–1469. 10.1016/j.ajem.2014.09.006 25440231

[29] Khanyi HB, Naicker B. The use of point-of-care ultrasound in a regional emergency department in KwaZulu-Natal, South Africa. S Afr Fam Pract. 2021;63(1):5269. 10.4102/safp.v63i1.5269 PMC8424764 34476962

[30] Rees H, Katzenellenbogen J, Shabodien R, et al. The epidemiology of incomplete abortion in South Africa. National Incomplete Abortion Reference Group. S Afr Med J. 1997;87:432–437. 9254785

[31] Lombaard H, Adam S, Makin J, Sebola P. An audit of the initial resuscitation of severely ill patients presenting with septic incomplete miscarriages at a tertiary hospital in South Africa. BMC Pregnancy Childbirth. 2015;15(1):82. 10.1186/s12884-015-0510-7 25886596 PMC4384342

[32] National Department of Health, South Africa. Essential Drugs Programme. Hospital level (Adults) Standard Treatment Guidelines and Essential Medicines List. 5th ed. Pretoria: The National Department of Health; 2019.

[33] Bigrigg MA, Read MD. Management of women referred to early pregnancy assessment unit: Care and cost effectiveness. Br Med J. 1991;302(6776):577–579. 10.1136/bmj.302.6776.577 1902383 PMC1669428

